# Sensing a Sensor: Identifying the Mechanosensory Function of Primary Cilia

**DOI:** 10.3390/bios4010047

**Published:** 2014-03-13

**Authors:** Rahul M. Prasad, Xingjian Jin, Surya M. Nauli

**Affiliations:** 1 College of Pharmacy and Pharmaceutical Sciences, The University of Toledo, Toledo, OH 43614, USA; E-Mails: rprasad@rockets.utoledo.edu (R.M.P.); xingjian.jin@rockets.utoledo.edu (X.J.); 2 College of Medicine and Life Science, The University of Toledo, Toledo, OH 43614, USA

**Keywords:** cilia, calcium signaling, shear stress, cell culture, tissue, perfusion

## Abstract

Over the past decade, primary cilia have emerged as the premier means by which cells sense and transduce mechanical stimuli. Primary cilia are sensory organelles that have been shown to be vitally involved in the mechanosensation of urine in the renal nephron, bile in the hepatic biliary system, digestive fluid in the pancreatic duct, dentin in dental pulp, lacunocanalicular fluid in bone and cartilage, and blood in vasculature. The prevalence of primary cilia among mammalian cell types is matched by the tremendously varied disease states caused by both structural and functional defects in cilia. In the process of delineating the mechanisms behind these disease states, calcium fluorimetry has been widely utilized as a means of quantifying ciliary function to both fluid flow and pharmacological agents. In this review, we will discuss the approaches used in associating calcium levels to cilia function.

## 1. Introduction

**Figure 1:**
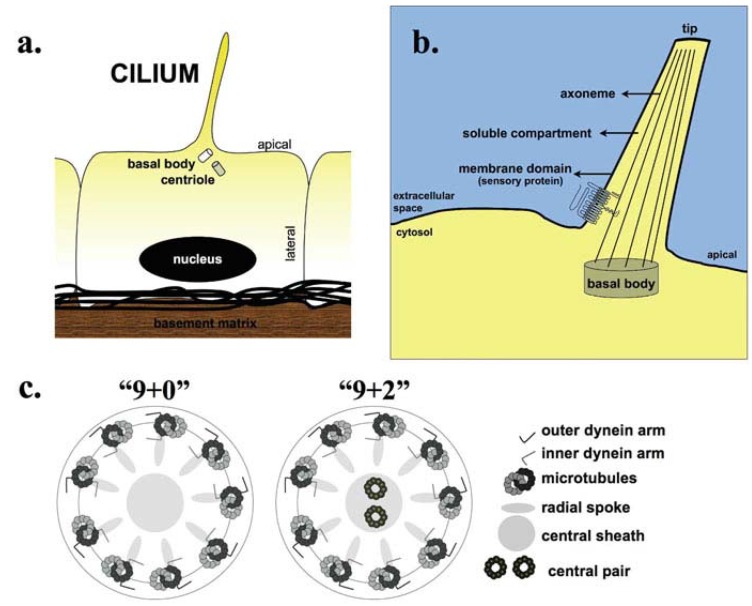
Structure of cilia. (**a**) Cilia are cellular organelles composed of microtubules, and they project from the apical surface of numerous cell types. (**b**) A cilium is composed of a membrane domain, a soluble compartment or cilioplasm, an axoneme and a basal body. The membrane domain contains multiple sensory and channel proteins, several of which play a role in calcium-mediated fluid-flow mechanosensation. (**c**) The orientation of the microtubules in the axoneme is categorized as “9+0” or “9+2” and is anchored to the basal body. Taken and adapted from [1].

Once enigmatic organelles, cilia have become a growing field of biomedical research. Cilia are often described based upon the structural arrangement of the microtubules of which they are composed, with the two types of classifications being “9+0” and “9+2”. In addition to the structure of cilia, the ability for independent movement or motility is another characteristic by which cilia are classified, with those capable of this function being termed “motile” and those incapable termed “non-motile.” Non-motile cilia with a 9+0 microtubule arrangement are often referred to as primary cilia (Figure 1). 

First described in 1898, primary cilia are found in a diverse group of cell types [2]. Over the past twenty years, cilia have emerged as ubiquitous organelles with a profound physiological impact. Indeed the growing list of disease states caused by ciliary defects has merited the use of the comprehensive term, “ciliopathies” (Table 1). 

Paralleling our understanding of the importance of cilia, delineating the specific functions of cilia has begun to gain momentum. Through a variety of functional assays, the primary cilium has been identified as a mechanical and chemical antenna that serves to translate extracellular stimulations to intracellular signals [3]. The mechanosensory function of cilia has been shown in renal epithelia, vascular endothelia, nodal cells, hepatocytes, pancreatic epithelial cells, osteocytes, chondrocytes, and odontoblasts [4,5,6,7,8,9,10,11].

During the sensing process, calcium plays an important role as a second messenger. This central paradigm has been developed by the findings of many independent groups showing that when challenged by cilia, specific mechano- or chemo-stimulations, an influx of intracellular calcium is often observed. This intracellular calcium-level fluctuation can be abolished in cells with dysfunctional primary cilia, and ultimately giving rise to ciliopathies. It is also noteworthy that primary cilia are loaded with a variety of calcium ion permeable channels, many of which, if mutated, could lead to abnormal intracellular calcium concentrations and subsequent cellular catastrophes. In this review, we discuss the three experimental approaches to observing and quantifying ciliary fluid-flow sensation: cell culture, single-cell, and *ex vivo* tissue perfusion assays.

**Table 1:** Ciliary classification, function and disease relevance in mammals. Taken and modified with permission from [1].

Axoneme	Motility	Function	Disease Relevance	Reference
“9+0”	Motile	Generation of nodal flow	Situs inversus; Situs ambiguous; Situs isomerism	[12,13,14]
	Non-motile	Sensation of nodal flow	Situs inversus; Situs ambiguous; Situs isomerism	[14,15]
		Mechanosensor	Polycystic Kidney, Liver, and Pancreas Diseases	[5,16,17]
		Shear stress sensor	Hypertension; Atherosclerosis; Aneurysm formation	[6,7,18,19]
		Osmolarity sensor	Respiratory diseases; Infertility	[20,21]
		Gravitational sensor	Osteoporosis; Chondroporosis	[22,23,24]
		Olfaction sensor	Anosmia; Hyposmia	[25,26]
		Light sensor	Retinitis pigmentosa; Blindness	[27,28,29]
		Chemosensor	Nephrocystin; Diabetes; Obesity	[30,31,32]
		Neurotransmitter sensor	Impaired brain plasticity	[33]
		Developmental regulator	Developmental defects; Cancer	[34,35,36]
		Pressure sensor	Bone maintenance, development	[22,37,38]
“9+2”	Motile	Chemosensor	Chronic obstructive pulmonary disease (COPD)	[39]
		Airway remodeling	Bronchiectasis; Hyperreactive airways	[40]
		Fluid Clearance	Chronic obstructive pulmonary disease (COPD)	[41,42,43]
	Non-motile	Oocyte Transport	Infertility	[44,45]
		Sperm Motility	Infertility	[46,47,48]
		Fluid Transport	Hydrocephalus; Cell Migration	[49,50,51]

## 2. Cell Culture Assay

**Figure 2:**
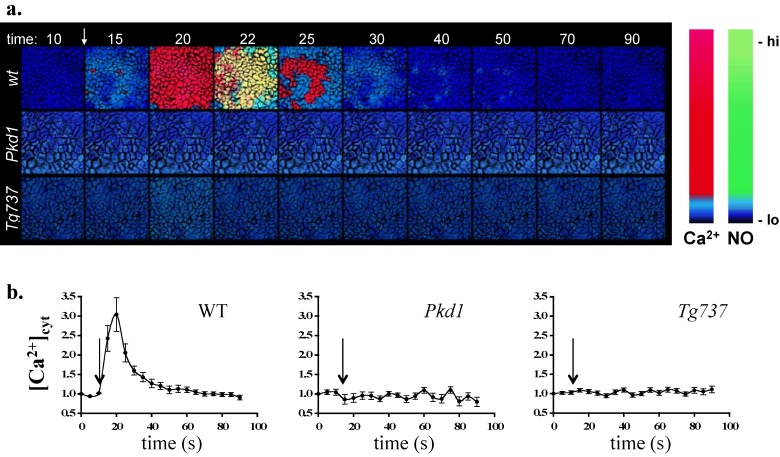
Calcium response to fluid flow in cell cultures. (**a**) The induction of fluid flow causes a rise in calcium in cells (pseudocolored). (**b**) In transgenic cell lines with knocked-out *Pkd1* or *Tg737*, both of which are ciliary proteins, calcium response is lost. The cells were incubated with fluorescent calcium dyes. Blue represents a low level; red denotes a higher level of calcium. Taken and adapted from [7].

Due to their widespread use, *in vitro* cell culture studies have provided impressive insight into the ciliary mechanosensation of fluid flow. The approach to conducting fluid flow cell culture assays is for the most part conserved: upon growing the cell types on a cytologically conducive surface, the cultured cell layers can be treated with a parallel fluid flow. The resulting shear-stress forces induce a rise in intracellular Ca^2+^ that can either be observed through transgenic calcium markers or fluorescent calcium dyes (Figure 2). In the case of fluorescent calcium dyes, the cell cultures must be incubated with the dye prior to induction of flow. 

In line with this overall approach, Nauli *et al*. details an experimental setup for growing cell monolayers, delivering fluid-shear stress, and observing the subsequent responses [52]. In the procedure, the authors describe several steps that are essential to the cell culture assay. The first such step is the presence of primary cilia to be induced and verified in the cell culture monolayer. As the primary cilia are only present in differentiated cells, one approach to inducing ciliation in, for example, kidney epithelial cells, is to grow cells overnight in normal serum and then in reduced serum (0.5%) for 48 h [52]. In addition to kidney epithelial and endothelial cells, other cell types can be utilized including embryonic cells, hepatocytes, pancreatic epithelial cells, osteocytes, chondrocytes, and odontoblasts [4,5,6,7,8,9,10,11]. 

In cell lines lacking transgenic calcium indicators, some of which have been established specifically for the ciliary domain, incubation with a fluorescent dye allows for visualization and quantification of ciliary function [53]. While the standard dyes utilized are Fura-2AM or Fluo-2, other conventional calcium indicators that are described above can be used. Following incubation with the calcium indicator, the cell culture must be transferred to a flow-delivering apparatus that is positioned with a computer and fluorescent microscope. Through the use of imaging software, such as MetaMorph, image capture can be used to analyze changes in fluorescence before, during and after perfusion of the cell culture monolayer. The calcium response profile produced as a result of flow is distinctive. Within renal epithelial and endothelial cell types, shear stress has been shown to cause a transient calcium influx after 5 seconds [6,7,16,54]. However, the peaks of calcium in response to shear stress could extend to 10–20 s, as has been seen by independent laboratories [55,56,57].

## 3. Single Cell Assays

It has long been speculated that the primary cilium itself functions as an independent calcium signaling compartment that might initiate a serial of downstream events. To test this hypothesis, it becomes critical to monitor calcium signaling in a single cell and cilium to achieve optimal resolution. However, such a signal cell assay is very difficult to conduct, owing to the submicron diameter and flexible nature of cilia. Therefore, it is only until recently that several independent research groups reported their observations about calcium signaling within an individual cilium. 

In contrast to cell culture assays, which use fluorescent calcium dyes, the majority of single-cell studies have utilized genetics to construct genetically encoded calcium indicators (GECI) that are specific to the primary cilium of the cell. By linking such indictors to a guide sequence that targets the primary cilium, researchers are able to dissect the calcium signals within an individual cilium from that of cytoplasm. Su *et al*. conducted a well-designed comparison experiment wherein several different GECI are fused with different cilia targeting sequences [58]. Parameters such as targeting efficiency, calcium signal dynamic range and cellular toxicity are carefully measured (Table 2).

**Table 2:** Comparison of genetically engineered calcium indicators. Data from Su *et al.* [58].

Fluorescent Indicator	Targeting Efficiency	Cytosolic Signal Dynamic Range (Maximum Percent Change in Fluorescence or FRET [SEM])	Ciliary Signal Dynamic Range (Maximum Percent Change in Fluorescence or FRET [SEM])
GFP	0%	-	-
5HT_6_-GFP	90%	-	-
5HT_6_-GCaMP5G	0%	65.1% [7.9%]	
5HT_6_-YC3.60	80%	58.2% [11.9%]	74.6% [8.8%]
5HT_6_-G-GECO1.0	75%	135.1% [42.4%]	360.0% [62.1%]
IA-GECO1.0	65%	175.6% [42.3%]	443.1 [39.5%]

This screening allows researchers to select a combination of GECI and cilia targeting sequences that can produce the highest signal definition and minimal cilia growth interferences. Ionomycin, ATP and fluid flow were applied to cells transfected with optimal GECI construct, and the subsequent responses were monitored and recorded by a confocal microscope system. In regard to fluid flow, a serial of z-stacking frames with a thickness of about 2 microns were continuously acquired along the base of a cilium. Once the cilium was bent, the whole image of a cilium could be captured. Also, the raw calcium signal readouts were normalized by an endogenous non-GECI fluorescent signal, such as mCherry, so that signal changes due to focal plain alternation could be eliminated. They reported cilia-specific calcium responses following fluid flow, echoing many previous reports that considered fluid flow as a cilia-specific stimulation. 

Similar to this investigation, Markus Delling *et al.* also studied cilia-specific calcium signaling using GECI and a confocal microscope [53]. Differently, they challenged the cilia with a laser pulse that could rupture the ciliary membrane and induce calcium influx localized to cilia specifically. It is very interesting to note that the calcium fluctuation following the rupture didn’t seem to be transduced to cytoplasm. Such highly localized calcium signaling was also related to the hedgehog pathway. This finding is different than some other reports that proposed a calcium-induced calcium release response that relayed ciliary calcium signaling to that of cytoplasm. In addition, Markus Delling *et al.* investigated the identity of calcium channels in an accompanying report [59]. Their finds show that the PKD1L1-PKD2L1 heteromeric channel establishes the cilium as a unique calcium compartment that modulates established hedgehog pathways. An important point is that this study did not investigate cilia response to fluid flow, which is thought to be the most physiologically significant stimulation to primary cilia.

**Figure 3:**
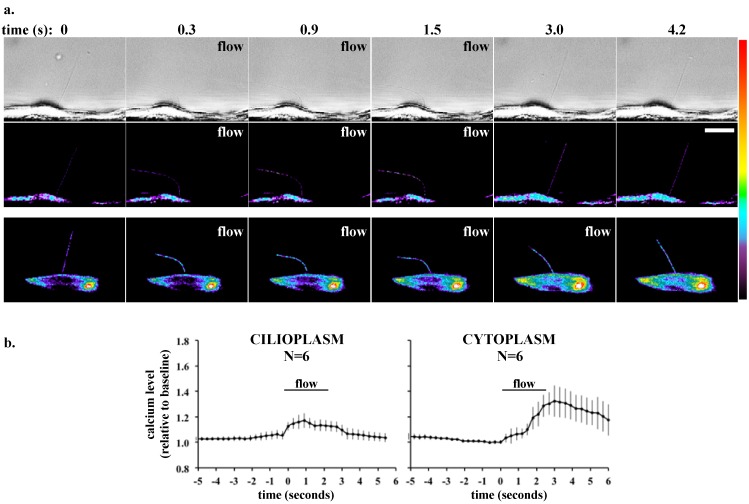
Ciliary calcium response to fluid flow in a single cell. (**a**) The calcium responses within the ciliary and cytoplasmic compartments of cells (pseudocolored) can be observed utilizing fluorescent calcium dyes or cell lines containing transgenic calcium indicators. (**b**) The induction of fluid flow causes bending of the primary cilium and a subsequent influx of calcium, first through the cilioplasm and then into the cell body. Color bar indicates calcium level, where black–purple and yellow–red colors represent low and high calcium levels, respectively. Bar = 4 μm. Taken and adapted from [60].

Xingjian Jin *et al.* utilized GECI in their study but, different from the aforementioned studies, they made use of a novel cell culture system [60]. Cells that carry GECI construct were cultured on a glass coverslip-based flexible substrate. When cells reached 100% confluence and were serum starved overnight, the substrate was peeled off the coverslip and placed diagonally on a string of microwire. This microwire coated with the substrate was then transferred to a regular fluorescent microscope equipped with cell culture chamber and special prism. By focusing on the edge of microwire, they were able to obtain a side view of cells growing on the substrate (Figure 3). 

One major advantage of this method compared to the previous studies is that the whole cilium always remained in focus, with no cytoplasmic background, which made it much easier and more accurate to differentiate calcium responses of cilium and those of cytoplasm. Using these systems, the authors showed cilia calcium responses to stimulations including fluid flow, fenoldopam and ionomycin. By applying different inhibitions, two calcium channels, Polycystin-2 (PC2) and L-type calcium channel (Cav 1.2), were found to be responsible for ciliary calcium responses. Intriguingly, calcium signaling initiated by these two channels seemed to have different fates, the former transduced to cytoplasm and the latter confined within cilium. Such differences, along with the findings made by Markus Delling *et al.*, suggest that the calcium transduction from cilium to cytoplasm could be controlled by an unknown machinery that can switch on and off the calcium transfer between cilium and cytoplasm in response to different initial stimulations [53]. 

Although a major breakthrough in single cilium calcium assay has been made the past year, there are still technical issues remaining to be solved. Firstly, all the GECIs utilized in the above studies were not ratiometric or semi-ratiometric. They are more comparable to Fluo-4 than Fura-2. Therefore, inaccuracies caused by random and uneven distribution of GECIs are inevitable. Secondly, the optimal expression levels of GECIs were not quantified in these studies, which might make it hard to reproduce such assays under other conditions. Thirdly, as for cell types that have very short primary cilia, such as osteoblasts and endothelial cells, it is still extremely difficult to visualize calcium signaling in their cilia. Considering how important ciliary calcium signaling is to these cell types, it becomes very urgent to develop a GECI construct that appears brighter at the baseline level combining with an imaging system that enables higher resolution. It is worth mentioning that the most direct way to study cilia as mechanosensory organelles is arguably by direct bending with a micropipette, an original technique introduced by Praetorius and Spring [61]. Despite the technical obstacles that remain to be solved, new methods that have enabled researchers to explore the significant and novel role of primary cilia as independent calcium signaling compartments are still exciting. Nevertheless, the discussed progress should serve as the significant first step to revealing more important roles of primary cilia in calcium signaling and related human diseases. 

## 4. *Ex Vivo* Assays

**Figure 4:**
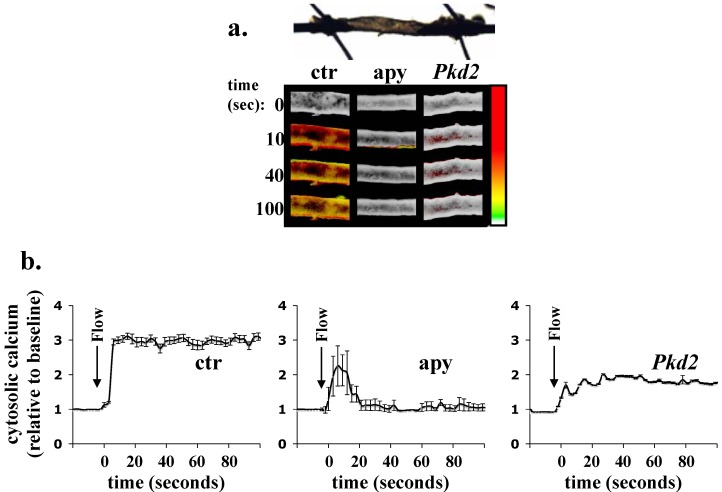
Calcium response to fluid flow in *ex vivo* arteries. (**a**) Within *ex vivo* arteries, fluid flow causes an influx of calcium. (**b**) In tissues with knocked-down *Pkd2*, a ciliary calcium channel, or that are treated with apyrase, the response profile is changed. Tissues were incubated with a fluorescent calcium dye prior to perfusion. Change in cytosolic calcium was pseudocolored; white/green represents a low level of cytosolic calcium, and yellow/red denotes a higher level. Taken and adapted from [6].

Analyzing tissue responses *ex vivo* is a powerful tool in overcoming the limitations of the cell culture approach to describing ciliary mechanosensation. Cell culture studies have provided numerous advancements in delineating the signaling pathways and mechanisms of *ciliary* fluid flow sensation. However, the nature of *in vitro* cell culture prevents a direct application of novel findings to physiologic models. *Ex vivo* studies provide an important bridge between applying cell culture findings in a more controlled, consistent, and efficient system than is possible with *in vivo* studies. The result is an approach that can be best utilized for confirming cell culture findings in a more physiological setting as well as for conducting high-throughput screens for pharmacological agents (Figure 4).

Observing ciliary mechanosensation *ex vivo* follows three experimental phases: tissue collection, preparation of tissue and flow system, and flow induction. While a variety of biological buffers have been utilized in perfusion systems, the selected buffer must be of constant pH, electrolyte, and O_2_/CO_2_ concentrations in order to ensure tissue viability. The fluid-flow assays have been developed for skeletal, renal, and cardiovascular tissues [62,63]. 

Ishihara *et al.* describe a method for observing shear stress-induced calcium within bone tissue. Utilizing embryonic chick tissue, they prepared bone fragments by first stripping away the periosteal layer and then trimming samples into 2 × 2 mm pieces, with a thickness between 60 to 80 μm. As with cell culture assays involving cells that lack a transgenic calcium marker, the tissue samples were incubated with fluorescent calcium dyes, such as fluo-8 and fura-2, in order to visualize intracellular calcium responses. The flow system utilized by Ishihara *et al.* uses capillary diffusion to apply fluid flow over the tissue sample [64]. The tissue is first placed on a glass slide that is held in place using adhesive grease, and the fluid flow is induced by adding a drop of solution to one side of the slide and suctioning the fluid from the opposite side using a piece of filter paper. As with all fluid flow calcium fluorimetry methods, a baseline is acquired prior to induction of flow. The osteocytes investigated by the group were identified within the tissue utilizing a three-dimensional analysis of the tissue layers [65]. Although Ishihara *et al.* do not specifically investigate primary cilia, presence and function of cilia have been identified in osteocytes [22,37].

The role of cilia in the pathogenesis of renal disease, namely polycystic kidney disease, emphasizes the need for applying cell culture findings to physiological systems in order to realize the potential for drug development. Liu *et al*. utilized *ex vivo* tubules from cortical collecting ducts in rabbits to characterize the function of cilia and, in the process, further consolidated paradigms of cilia as fluid flow sensors in the renal system [55]. Freehand isolation of the tubules was conducted, followed by microperfusion. The perfusion system involved cannulation of the isolated tubule, positioning of the tubule on the Cell-Tak painted coverslip within a specimen chamber filled with incubation media and, finally, perfusion of media via syringe pump [66]. As with previously mentioned methods, fluorescent calcium dyes were utilized to observe the intracellular responses. In contrast to incubating the tissue prior to flow system setup, as in Ishihara *et al.*, the calcium dye was added to the incubating media, followed by an incubation period prior to flow induction. In addition to perfusion of intact tubules, Liu *et al.* perfused tubules that had been cut longitudinally, thereby exposing the lumen, as well as intact tubules with occluded lumens. Their findings showed that cell types within the opened tissue had a different calcium response to intact tubules or occluded lumens, explained by the lack of circumferential stretch and subsequent signaling response when the lumen is opened and exposed [55]. The effect of circumferential stretch is an example of a nuance within the physiological system that is neglected in cell culture experiments. Worth mentioning is that fluid perfusion in renal tubules can also regulate acid–base balance in the kidney [67]. In addition, the primary cilia in macula densa cells within the cortical thick ascending limb can detect tubular fluid-flow rate, which can alter the glomerular filtration rate via the tubuloglomerular feedback pathway [68].

Studies on elicited calcium responses have been conducted not only in tubular renal tissues but also in vascular tissues. Abou-Alawi *et al.* conducted studies on *ex vivo* mouse aortae, simulating the effects of blood flow [6]. Similar to the studies conducted by Liu *et al.*, the dissected tissue was cannulated in a bath of media and then perfused with media. In contrast to other studies, the tissue was incubated in a solution containing the fluorescent calcium dye before cannulation and perfusion. Although the calcium response profiles in the *ex vivo* tissues were not identical to cell culture studies, reflecting the complexity of physiologic factors that limit cell culture studies, the knockdown of the ciliary calcium channel *Pkd2* did inhibit the response profile as it did in cell culture studies (Figure 4). The ability to reinforce novel cell culture findings using the *ex vivo* system, coupled with defined responses through calcium fluorimetry, is an integral step in the process of contributing to physiological models and developing clinical treatments for disease states.

As in the renal tubule, fluid flow can cause vessel distension, and this may not reflect a function of the primary cilia. Furthermore, the calcium profile is substantially different between the control vessel and the vessel treated with apyrase which is an enzyme that hydrolyzes any nucleoside triphosphates or diphosphates (such as ATP and ADP). It is very possible that the vessel distention induces secretion of ATP or ADP. In particular, it has been shown that ATP can increase calcium in the cilioplasm [58]. Thus, the vessel distention and the presence of ATP could undermine the strategy to study cilia function.

**Figure 5:**
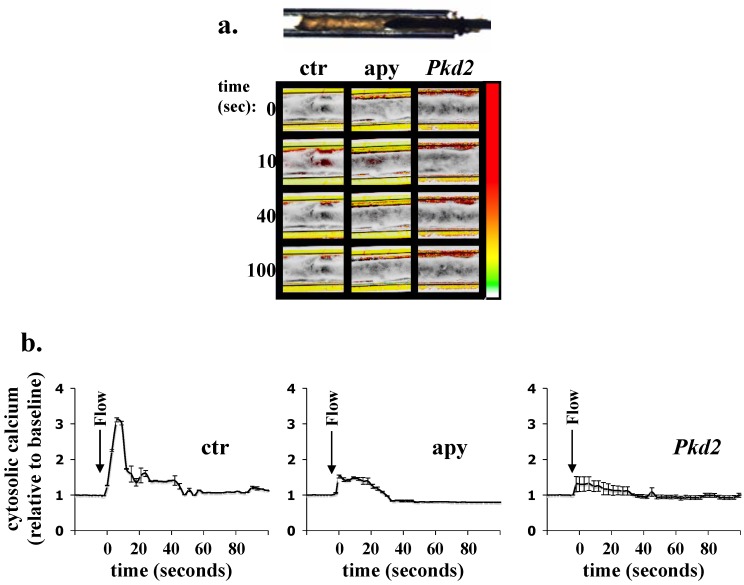
Calcium response to fluid flow in capillary-enclosed arteries *ex vivo*. (**a**) Within the capillary-enclosed arteries *ex vivo*, fluid flow causes an influx of calcium. (**b**) In tissues with knocked-down *Pkd2* or in tissues treated with apyrase, the response profile is changed. Tissues were incubated with a fluorescent calcium dye prior to perfusion. Change in cytosolic calcium was pseudocolored; white/green represents a low level, and yellow/red denotes a higher level of cytosolic calcium. Taken and adapted from [6].

To avoid these issues with vessel distention and ATP, AbouAlaiwi *et al.* have developed a technique to insert a vessel into a glass capillary tube (Figure 5). The basis of the approach is that the vessel inside the capillary tube would have no or very limited room for distention or expanding due to the perfusate pressure [6]. The calcium profiles between freely placed and capillary-enclosed vessels are indeed very different. Because the calcium profile of the capillary-enclosed artery represents a much closer resemblance to the profile of a cell culture, we believe the capillary-enclosed approach should be used to study mechanosensory cilia function.

Another exciting *ex vivo* assay to study cilia function is the embryonic nodal system [4,69,70]. Primitive streak and pit are formed during embryogenesis, where the upper layer of embryonic cells invaginates. The *Hensen’s node* located at the end of the primitive streak is most likely the earliest role of primary cilia that is currently known. This *ex vivo* model has allowed us to study different functions of primary cilia in sensing fluid shear stress and detecting chemical gradient. Responding to the nodal flow or chemical gradient, only the left-side margin of the node would show an increase in intracellular calcium, an early contributing factor to determining left–right body asymmetry. Thus, regardless of the mechano- and/or chemo-sensory roles of primary cilia, cytosolic calcium is an important indicator to validate cilia function within the *Hensen’s node*.

Despite the fact that calcium has been shown to play an essential role as a second messenger to transmit the extracellular signal into an intracellular biochemical reaction, it is also important to mention that some studies have indicated that calcium might not be involved in cellular mechanosensory transduction [22,71,72]. There is no doubt that the discrepancy in this area requires more research, through a better, more sensitive and innovative technology. However, the idea of primary cilia as sensory organelles seems to be generally accepted. Perhaps an *in vivo* system would help to provide a better understanding of the mechanosensory function of primary cilia in the near future.
